# Causes of Shoulder Dysfunction in Diabetic Patients: A Review of Literature

**DOI:** 10.3390/ijerph19106228

**Published:** 2022-05-20

**Authors:** Filip Struyf, Michel GCAM Mertens, Santiago Navarro-Ledesma

**Affiliations:** 1 Department of Rehabilitation Sciences and Physiotherapy/MOVANT, Faculty of Medicine and Health Sciences, University of Antwerp, Universiteitsplein 1, 2610 Wilrijk, Belgium; filip.struyf@uantwerpen.be (F.S.); michel.mertens@uantwerpen.be (M.G.M.); 2 Department of Physiotherapy, Faculty of Health Sciences, University of Granada, Campus of Melilla, Querol Street 5, 52004 Melilla, Spain

**Keywords:** diabetes mellitus, type 2, shoulder adhesive capsulitis, shoulder injuries, hyperglycemia, systemic review

## Abstract

Objective: Understanding the underlying mechanisms behind shoulder dysfunctions in patients with diabetes mellitus. Study Design: Systematic qualitative literature review. Participants: Patients with shoulder dysfunctions and diagnosed with impaired glucose tolerance or diabetes mellitus. Intervention: Published scientific literature containing evidence about the mechanisms of shoulder dysfunctions in the diabetic population. Articles were selected based on criteria containing diabetic population, shoulder dysfunction, methodological quality ≥ 6/9 and >20 subjects. Main Outcome measures: range of motion; questionnaires (QoL, UCLA-m, SPADI, DASH); blood glucose, blood HbA1C; calculated capsular stiffness (Kcap); calcification shoulder joint; shoulder dysfunction in patients with glucose metabolism disorders and diabetes mellitus. Results: We found 17 published articles with level 2 and 3 evidence. Multiple factors such as age, duration of diabetes mellitus (DM), glycated hemoglobin (HbA1c), advanced glycation end products (AGE), vascular endothelial growth factor (VEGF), interleukin-1ß (IL-1ß) were shown to be associated with tendon changes and increased capsular stiffness (Kcap) conceivably leading to limited range of motion (ROM) or frozen shoulder. Decreased ROM and frozen shoulder have a significantly higher prevalence in DM than in non-DM. Conclusions: Based on the current literature we confirm a high prevalence of shoulder dysfunctions in patients with diabetes mellitus. The cause of the shoulder complications is unknown, and more research is mandatory to shed more light on the complex interplay between the multifactorial causes of shoulder dysfunction in diabetes mellitus.

## 1. Introduction

Diabetes mellitus (DM) type 2 is a chronic disease characterized by elevated levels of blood glucose over a prolonged period of time [1]. If left untreated, diabetes can cause serious long-term complications to the vascular and nervous system [2]. DM is typically preceded by a state of impaired fasting glucose and/or impaired glucose tolerance which may cause damage to many bodily systems immediately after its presence [3]. DM is also associated with reduced life expectancy, and significant morbidity due to specific microvascular complications (retinopathy, nephropathy and neuropathy), macrovascular complications (ischemic heart disease, stroke and peripheral vascular disease), and a reduced quality of life [4]. Based on a recent systematic review, it has been estimated that the direct annual cost of DM worldwide is beyond (US) $827 billion.

Interestingly, patients with DM report twice as much musculoskeletal complaints in comparison to age and gender matched healthy controls [5]. Despite the increased prevalence and its social impact, musculoskeletal disorders are less well studied in comparison to other diabetic complications [6]. Advanced age, longer duration of DM, and hypertension have been reported as risk factor for musculoskeletal disorders in DM [7]. For instance, Cole et al. reported that over one-quarter of patients with diagnosed DM have shoulder complications and the incidence of frozen shoulder has been shown to range from 10 to 35% [8,9,10]. The most frequent reported shoulder problem in DM is indeed frozen shoulder, also referred to as adhesive capsulitis [7,8,9,10]. Although the exact etiology of musculoskeletal disorders in general and adhesive capsulitis in particular, is relatively unknown, accumulated and irreversible crosslinks between adjacent protein molecules, damaged vessels and nerves, and an increase of collagen in connective tissue are among the suggested underlying pathogenic factors that may contribute to the development of musculoskeletal disorders in DM patients [7,8]. Evidence for the above-mentioned pathogenic pathway that may cause shoulder dysfunction in DM comes from animal studies that reported an increase in tendon diameter and tendon stiffness in diabetic mice [11]. The underlying mechanism that leads to tendon dysfunction is inflammation. Inflammatory mediators such as tumor necrosis factor alpha (TNF-α) and interleukin-6 (IL-6) are chronically released in DM subjects and in turn lead to a cascade of inflammatory reactions causing chronic inflammation, buildup of collagen and other extracellular matrix components which, in the end, may result in fibrosis [12,13,14,15]. The increase in stiffness in connective tissue caused by the microstructural reorganization of collagen fibers that causes adhesive capsulitis is linked to the preceded development of advanced glycation end-products [16,17,18,19,20,21].

There is an increasing need of information in primary care rehabilitation about possible comorbidities and the related etiology of certain patient groups. For instance, understanding of the mechanisms in DM associated musculoskeletal dysfunctions may give rise to alternative and more effective therapeutic approaches. Due to its importance and its relatively unfamiliarity in clinical use, this present review aims at systematically investigating musculoskeletal shoulder dysfunction in DM patients. Our research questions were:

1. Are the higher amounts of glycated hemoglobin, indicating poorer control of blood glucose levels, associated with shoulder dysfunction in type 2 DM patients?
2. What are the mechanisms behind the increased incidence of shoulder complaints in type 2 DM patients?

## 2. Methods

This review was conducted in accordance with the PRISMA guidelines, which is an updated statement addressing the conceptual and methodological issues of the original QUOROM statement [22].

### 2.1. Eligibility Criteria

Eligibility criteria were used according to the PICOS strategy [23]. PICO(S) components (**P**atients, **I**ntervention, **C**ontrol and **O**utcome) were used to establish the research problem. Eligibility assessment of the search results was performed according to specific in- and exclusion criteria set.

Inclusion criteria were:

1. **P**: subjects with a glucose metabolism disorder
2. **I**: Randomized Controlled Trial, case control, cohort, and cross-sectional studies
3. **C**: compared with a non-type 2 DM control group
4. **O**: association between metabolic derangements and shoulder dysfunction, full text written in English, Dutch, German, or French,

Exclusion criteria were:

1. diseased (acute non metabolic disease),
2. children (<10 years of age) and geriatric population (>65 years of age)
3. languages other than mentioned above and
4. meta-analysis, systematic reviews.

### 2.2. Information Sourcesand Search Strategy

A literature search for studies that identified the mechanism behind the development of a musculoskeletal disorders, more specific shoulder dysfunction, in the type 2 DM population was conducted till December 2022. Articles were identified by searching the electronic databases PubMed and Web Of Science (WOS). Keywords were derived from the PICOS-items and were converted into Mesh-terms and free-text words. The search resulted in 436 hits in Web Of Science and 498 hits in PubMed. After removing duplicates, 634 articles remained. In addition, we manually searched for relevant articles based on article citations and reference lists. The date of the last search by two of the authors (FS and SNL) was performed in December 2022.

To ensure compatibility towards all databases, Medical Subject Headings (MeSH) used in PubMed were changed into free keywords. The following MeSH-terms were used:

*For PubMed*: (“Diabetes Mellitus” [Mesh] OR diabetes OR “blood glucose” OR “blood sugar” OR “glucose metabolism” OR “Blood Glucose” [Mesh] OR “Glycosylation End Products, Advanced” [Mesh] OR glycosylation) AND shoulder AND (“Tendinopathy” [Mesh] OR tendinopathies’ OR tendinopathy OR tenosynovitis OR tendinitis OR tendonitis OR tendonitides OR tendinosis OR “Bursitis” [Mesh] OR bursitis OR bursitides OR adhesive capsulitis OR “Frozen Shoulder” OR “Myositis” [Mesh] OR myositis OR myositides OR myopathy OR “Muscle Diseases” OR “Musculoskeletal Pain” [Mesh] OR musculoskeletal pain OR “Muscle Weakness” [Mesh] OR “muscle weakness” OR “muscular weakness” OR “Synovitis” [Mesh] OR synovitis OR synovitides OR “Rotator Cuff” [Mesh] OR rotator cuff OR “Shoulder Impingement Syndrome” [Mesh] OR impingement OR slap OR “Calcinosis” [Mesh] OR calcinosis OR “Arthritis” [Mesh] OR arthritis OR “Shoulder-Hand Syndrome” OR “Reflex sympathetic dystrophy” OR “Osteoporosis” [Mesh] OR osteoporosis OR dysfunction OR disease OR disorder OR disability). No filters were used.

*For WOS*: ((“Diabetes Mellitus” OR diabetes OR “blood glucose” OR “blood sugar” OR “glucose metabolism” OR “Blood Glucose” OR “Glycosylation End Products” OR glycosylation) AND shoulder AND (“Tendinopathy” OR tendinopathies’ OR tendinopathy OR tenosynovitis OR tendinitis OR tendonitis OR tendonitides OR tendinosis OR “Bursitis” OR bursitis OR bursitides OR adhesive capsulitis OR “Frozen Shoulder” OR “Myositis” OR myositis OR myositides OR myopathy OR “Muscle Diseases” OR “Musculoskeletal Pain” OR musculoskeletal pain OR “Muscle Weakness” OR “muscle weakness” OR “muscular weakness” OR “Synovitis” OR synovitis OR synovitides OR “Rotator Cuff” OR rotator cuff OR “Shoulder Impingement Syndrome” OR impingement OR slap OR “Calcinosis” OR calcinosis OR “Arthritis” OR arthritis OR “Shoulder-Hand Syndrome” OR “Reflex sympathetic dystrophy” OR “Osteoporosis” OR osteoporosis OR dysfunction OR disease OR disorder OR disability)). No filters were used.

### 2.3. Data Extraction and Analysis

Two researchers independently performed data extraction by selecting relevant data and integrating them into two separate databases. The two databases were compared and integrated into a final extraction table. Again, disagreements were resolved through discussion and by using a third investigator. None of the authors were contacted for further information when information was missing or unclearly reported. 

### 2.4. Assessment of Risk of Bias across Studies

Methodological quality assessment of the selected papers was performed using two assessment scales. First, case controls were scored using the Newcastle Ottawa quality assessment Scale (NOS) [24]. The checklist consisted of eight main items (case definition, representativeness of the cases, selection of controls, definition of controls, comparability, ascertainment of exposure, same method of ascertainment, non-response rate). The sub-items were scored and a total of nine points could be obtained. A high score indicated the study was designed and conducted in a manner that minimized the risk of bias. A priori, we decided that studies with less than six points would be excluded from this review based on low methodological quality. Second, cross-sectional studies were screened combining the design specific criteria described in the AHRQ to assess the risk of bias for benefits for cross-sectional studies [25]. Selection bias was unlikely in our review because of the large search strategy without exclusion on basis of severity of disease/publication date/origin of population. Patients with chronic conditions, rather than those who are asymptomatic, however, are more likely to participate in such studies. Therefore, reporting bias could be present. We chose to extract specific outcomes out of the studies, and thereby not present results that do not fit in any of our subgroups.

### 2.5. Data Items

From the included papers, the information was extracted and presented in an evidence table (Table 1). The following study characteristics were systematically extracted: bibliographic reference, study design, patient characteristics (including number, age, gender, and medical condition), outcome measures and results. Both authors extracted the data independently, after which the data were merged.

## 3. Results

### 3.1. Study Selection

**Figure 1:**
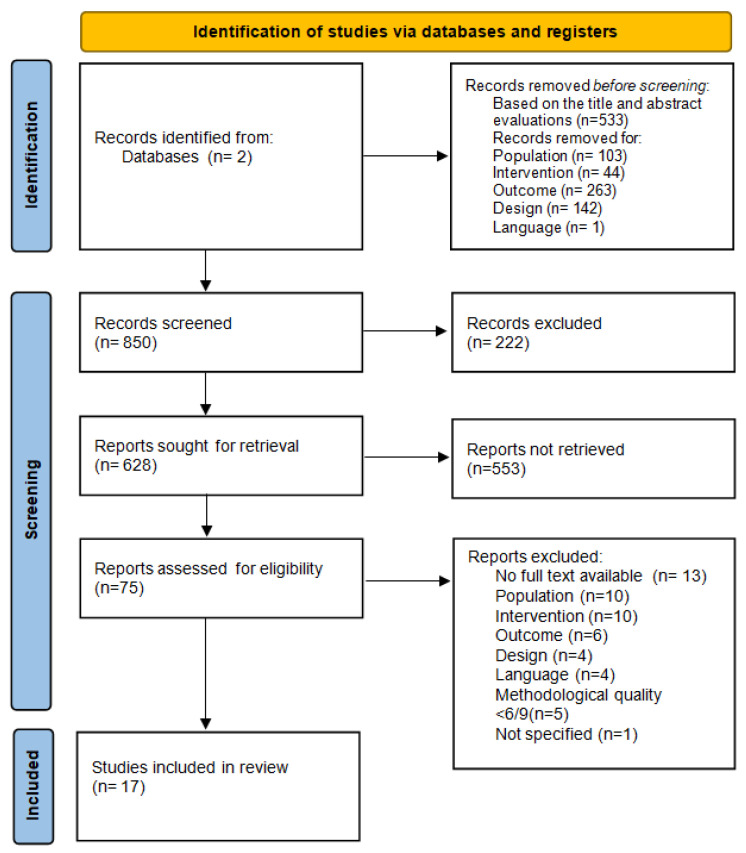
PRISMA screening process for selection of articles for review.

The literature search resulted in a total of 850 studies. After removing duplicates, 628 articles remained (Figure 1). In this case, 22 remaining studies were considered eligible and were scored for their methodological quality and risk of bias (Table 2 and Table 3). Five articles had to be excluded based on low methodological quality (<6/9). Ultimately, 17 articles remained available for our review.

### 3.2. Risk of Bias within Studies

Quality assessment was performed. Studies with a score higher or equal to 6/9 were included. The strength of the evidence provided by an individual study depends on the ability of the study design to minimize the possibility of bias and to maximize attribution. Following the hierarchy of study designs, the case-controls were given a “B” quality; cross-sectional studies were rated as “C” (Table 1 and Table 2). After these scores were given, it was possible to determine the level of evidence (1–4) (Table 3).

### 3.3. Risk of Bias across Studies

The methodological quality scores are presented in Table 1 and Table 2. Scores lower than 6/9 were excluded. The main score of the 17 remaining articles was 7.5/9.

### 3.4. Risk of Bias in Individual Studies

Methodological quality assessment is showed in Table 2 and Table 3. 

Screening of title, abstract, and full text, as well as the assessment of the methodological quality of the studies, was independently performed by the two researchers. Disagreements between reviewers were resolved by consensus with a third investigator. For the quality assessment, consensus had to be reached on each sub-item. In most cases, the two researchers agreed (91% or 180 of the 198 items). After a second review and a comparison of the 18 differences, the reviewers reached a consensus for all items. There was no need of a third opinion because of the full agreement. Methodological quality was varying with scores between 2/9 and 9/9. Four studies reached the total amount of points. We chose to include only articles with scores equal or higher to 6/9. In total, 17 included studies consisted of 151,528 subjects, of which 48% percent women.

### 3.5. Synthesis of Results

From the 17 included articles we were able to extract 6 different subgroups. The subgroups were shoulder ROM, questionnaires, medical imaging, blood samples, synovial samples and a group describing chronic complications of DM. The results of every single article were divided and placed under each relevant subgroup. Nine articles could be cited under “ROM”, five under “questionnaires”, five articles under “medical imaging”, nine articles under “blood sample”, three under “synovium sample”, and finally four under “chronic complications of DM”. Results were systematically merged to acquirea clear synthesis.

### 3.6. Results of Individual Studies

#### 3.6.1. Shoulder Range of Motion (SROM)

In particular, shoulder abduction [8,26,28,32,37,40] flexion [8,26,29,32,37,38,39,40], external rotation [28,32,37,40], internal rotation [28,32,40], extension [37] and scapular upward rotation [39] were evaluated. Methods used for measuring SROM were goniometry [26,32,37,40], inclinometry [8] and 3D tracking devices [38,39].

In general, SROM in patients with DM was significantly reduced in all of the above-mentioned movements [8,26,37,38,39,40], except for scapular upward rotation [39] in comparison to non-DM patients. Although age is a factor that naturally decreases ROM [26,28] the difference between DM and non-DM remained after statistically controlling for age [26]. Furthermore, Lee et al. [32], showed that capsular stiffness (Kcap), as measured by calculating the slope of the elastic phase in pressure-volume curves, in frozen shoulder patients was negatively correlated with SROM but no difference in Kcap between DM and non-DM could be found.

The relation between SROM and the duration of DM revealed conflicting results. One study reported no relation with SROM [26] and 2 studies suggest there was a correlation with age of patients, duration of diabetes, neuropathy, and the other hands’ problems [28,37]. Finally, the relation with medication use showed that individuals using insulin had an improved active flexion than those on diet and oral hypoglycemic agents alone [29].

#### 3.6.2. Questionnaires

To determine different aspects of shoulder disability the following questionnaires were used: SPADI (Shoulder pain and disability index) [8,39], UCLA-m (Modified University of California at Los Angeles Shoulder Rating Scale) [29], HAQ (health assessment questionnaire) [35], GALS screening examination (Girdle, arms, legs and spine) and DASH (Disability of arm, shoulder and hand) [38,39].

DM patients demonstrated more shoulder pain and a higher level of disability and reported more difficulties in performing activities of daily living [29,35,38,39]. Cole et al. reported no significant difference in prevalence of shoulder pain/stiffness between DM and non-DM after adjustment for age, sex, smoking and obesity [8].

#### 3.6.3. Medical Imaging

Medical imaging techniques used to study the tendons of the supraspinatus- [26,27,31,38], infraspinatus [27,31], long head of biceps [27,31,38], and subscapularis [27,31], and the non-specified rotator cuff [40] and sub-acromial-subdeltoid bursa [27] were: ultrasonography [27,31,39,40] and Magnetic Resonance Imaging [40].

DM was associated with degenerative changes in shoulder tendons in asymptomatic subjects [26,27,38]. Supraspinatus tendon-and long head of biceps tendon thickness was significantly higher (due to abnormal storage of collagen layers) in DMthan in controls [27,38]. Tenosynovitis of the long head of the biceps tendon and sub-acromial-subdeltoid bursitis were more frequently seen in DM [27]. One study showed that major lesions in the supraspinatus tendon were seen more frequently in diabetics than in healthy controls [26], and in a case control the difference was only significant for minor tears of the supraspinatus tendon [27]. No difference in tear type of the rotator cuff between DM and non-DM was reported [40]. In symptomatic subjects, the ratio of rotator cuff tears and calcifying tendinopathy was not different between DM patients and non-DM, although significantly higher symptomatic bilateral shoulder problems were found in DM [31].

Taking the duration from the onset of DM into account, no differences were found in size of lacerations in the supraspinatus- and long head of biceps tendon [27]. However, four studies did not take duration of DM into account [26,31,38,40].

#### 3.6.4. Blood Analysis

DM patients with frozen shoulders had significantly higher plasma levels of cholesterol and triglyceride compared to DM patients without frozen shoulders and controls [34,36]. No significant relation between frozen shoulders and any of the serum values urea, uric acid, creatinine, calcium, or phosphorus could be found [28,33,34], except for a significantly lower magnesium concentrations in comparison with DM patients without frozen shoulders [34]. Balci et al. [28] found no relation between frozen shoulders and plasma triglycerides, total cholesterol concentration and HDL- and LDL cholesterol concentrations [28].

High HbA1c levels, a reliable measure of long-term increased blood glucose concentrations, were associated with significant limitations in SROM [8], but not, after adjustment for age, gender, obesity and current smoking, with shoulder pain/stiffness [8], frozen shoulder [33] or any of the UCLA-m scores [29]. Fasting blood glucose was not associated with any of the UCLA-m scores [29] but was positively correlated with the incidence of frozen shoulder [36]. Finally, HbA1c levels was not associated with frozen shoulders [46].

#### 3.6.5. Synovial Fluid

Two studies reported on the analysis of synovial fluid from the sub-acromial-subdeltoid bursa. Vascular Endothelial Growth Factor (VEGF) and IL-1ß levels were studied [30,40]. Handa et al. showed that VEGF mRNA was expressed in the majority of patients with rotator cuff disease (51 out of 67) and was 100% positively expressed in DM patients [30]. The VEGF isoform VEGF121 was expressed in all diabetics, and the isoform VEGF165 in 12 out of the 14 diabetics. No significant expression of VEGF 189 and VEGF206 was found.

In addition, significantly higher subacromial IL-1ß levels were reported in DM patients with rotator cuff tearing [40]. The IL-1ß concentrations were significantly correlated with reduced SROM [40].

#### 3.6.6. Chronic Complications of DM

Different results were published regarding chronic complications in DM and shoulder complaints. Two studies reported an association between locomotor manifestations and the presence of other chronic complications of DM [28,35,37,46]. The results of neuropathy and SROM in the shoulder was inconsistent [28,37]. Similar results were reported for frozen shoulders in DM except for retinopathy [28].

## 4. Discussion

We have conducted a comprehensive systematic review of studies addressing the causes of shoulder dysfunction in diabetics. Our findings show that based on the current literature the underlying causes of shoulder dysfunctions in patients with diabetes mellitus remains relatively unknown.

Diabetes Mellitus (DM) is a metabolic disease characterized by hyperglycemia that, eventually results in chronic damage and dysfunction of multiple organs [47]. Recently, a meta-analysis on the association between the metabolic diseases and osteoarthritis indicated the extensive devastating effects of the complications in metabolic diseases on musculoskeletal organs [48]. Shoulder range of motion (SROM), not only frozen shoulders, is an important parameter for overall shoulder function and has been shown to be reduced in diabetic subjects compared to age-matched healthy controls [5,8,10,26,37,39]. It is reasonable to hypothesize that the reduced SROM in diabetics is caused by structural changes in the glenohumeral joint which themselves is caused by the long-term metabolic derangements as seen in DM. Surprisingly, capsular stiffness, an important marker for shoulder function and expressed as Kcap, was not different between DM patients and non-DM patients [32]. These findings indicate that Kcapis not the only determinant for a limited SROM, in diabetics [32]. For instance, there is evidence that degenerative changes and increased body of the supraspinatus-/biceps tendon exists in asymptomatic DM subjects [26,31,37]. Confirmation for the latter findings comes from animal studies that showed that hyperglycemia resulted in a reduction of proteoglycan levels caused by a decreased synthesis of glycosaminoglycans, which, in turn, may contribute to the tendon pathology as reported in DM [49]. Another study in rats showed that isolated hyperglycemia induces a chronic inflammatory response and consequently changes in the structure of the tendon [50]. In this context, a recent review presents a systemic view on the pathogenesis of frozen shoulder, including insulin resistance, low-grade inflammation, and chronic hypoxia. These mechanisms, together with the influence of modern life, including a sedentary lifestyle, and the partial or complete absence of range of motion of the shoulders in general and of the non-dominant shoulder, suggest that the pathology of FS is systemic [51,52].

There are conflicting results concerning the type and/or number of rotator-cuff tears and only weak evidence on the number of rotator-cuff tears and calcifying tendinopathies between DM and non-DM subjects, so no final conclusions can be drawn regarding rotator-cuff tears in asymptomatic subjects [31,39].

Since most shoulder problems in DM patients are of bilaterally origin [26,27,31,32,33,36], a systemic factor that could explain the underlying etiology of shoulder problems should be considered.

Chronically increased blood glucose level, as measured by an increase in HbA1c, could play an important role in the high prevalence of shoulder- and other musculoskeletal disorders in DM. Conflicting evidence, however, was found between the prevalence of shoulder problems and HbA1c levels. Some studies demonstrate an increased prevalence of shoulder dysfunction, frozen shoulder and reduced SROM associated to elevated HbA1c levels [8,35,36,37], whereas other studies showed contradictory results [33,53]. A remarkable finding are the differences in musculoskeletal abnormalities between type 1 and type 2 diabetics [35]. Although HBA1c levels were equivalent in type 1 and type 2 DM, frozen shoulder was significantly more prevalent in type 2 DM [51,52]. According to the latter results, an increase in HbA1c cannot be considered and independent contributing factor to the higher incidence of FS in DM. Other factors, such as lower socio-economic levels have been suggested to play a role but evidence for such assumptions is lacking [29]. There are indications that decreased plasma magnesium levels are associated with frozen shoulder in diabetics [34], as well as inflammatory lipoproteinemia’s, particularly hyper low-density lipoproteinemia and hyper non high-density lipoproteinemia, are associated with adhesive capsulitis accompanied by diabetes [34], whereas other plasma constituents, such as urea, uric acid, creatinine, calcium and phosphorus are not [28,33,34].

There is a relationship between the production of vascular endothelial growth factor isoforms (VEGF-121 and 165) and the development of shoulder joint contractures in type 2 DM with rotator cuff disease [30].

Furthermore, high levels of Advanced Glycation End (AGE) products have been associated with increased biceps tendon thickness and reduced shoulder flexion and have been shown to be significantly different in DM [38]. However, the existing evidence specific for shoulder problems in DM is weak and indirect and needs more scientific attention [54]. Furthermore, it has recently become clear that environmental factors such as diet, lack of exercise and smoking play an important role in the formation AGE products [55,56].

Chronicity is a hallmark of DM. It is conceivable that the decrease in SROM observed in many DM patients are parallel processes of aging and an accelerated muscle skeletal degeneration. Support for this hypothesis comes from a study suggesting DM has an additive negative effect on limited joint mobility, but no significant correlation with the duration of DM [26]. In addition, Cole et al. found no significant difference in prevalence of shoulder pain/stiffness between DM and non-DM subjects after adjustment for age, sex, smoking and obesity [8].

Interestingly, a recent study has shown an association between fasting glucose levels and adhesive capsulitis in a normoglycemic population, concluding that AC is positively associated with fasting glucose levels of 90–99 mg/dL, which are currently considered normoglycemic. This may indicate the importance of taking the glucose metabolism into account when assessing shoulder dysfunction or a potential adhesive capsulitis development as well as proposing new strategies in the treatment of shoulder conditions [57].

### Limitations

Confounding factors such as age, sex, weight, duration of DM, insulin use, and physical activity might have influenced the results of our included studies. Since most type 2 DM patients are obese and since obesity effects many biochemical blood parameters, it is remarkable to notice that only one out of the nine included studies adjusted for obesity or obesity related side-effects [8].

Finally, we wish to mention that the retrospective nature of several included articles has some disadvantages as well. Incomplete data or incorrect data, lack of reporting and the use of unconfirmed information might have led to reduced quality of the studies. The inclusion of both case control and cross-sectional designs, made it hard to compare the results.

## 5. Conclusions

Based on the current literature we confirm a high prevalence of shoulder dysfunctions in patients with diabetes mellitus. However, the underlying causes of the shoulder complications remain largely unknown. Although fasting blood glucose and HbA1c concentrations are serious candidates for the molecular derangements of/in the glenohumeral joint more research is mandatory to shed more light on the complex interplay between the multifactorial causes of shoulder dysfunction in diabetes mellitus.

## Figures and Tables

**Table 1:** Characteristics of the selected studies.

Title	Design	Sample	Outcome	Results
Abate et al. (2011) [26]	Case control	Group 1: 30 subjects with NIDDM type Il and good glycemic control (age 73.9 ± 12.72) Group 2: 30 subjects without DM matched for age and gender (159 age 74.3 ± 4.24) group 3: 10 normal young subjects (age 26.3 ± 1.6)	/a/ROM (goniometer) US evaluation SST	ROM (ABD & FL) reduced in both group 1 and 2 vs. 3 (*p* < 0.001); ROM (ABD & FL) reduced in group 1 vs. 2 (*p* < 0.001). More US abnormalities in group 1 vs. 2.
Abate et al. (2010) [27]	Case control	Group 1: 48 asymptomatic subjects with NIDDM type Il (age 71.5 ± 4.8) Group 2: 32 asymptomatic subjects, matched for age and sex, without NIDDM (age 70.7 ± 4.5)	US evaluation SST, IST, SScT, BT and SAD	SST and BT thickness greater in DM group (*p* < 0.001). More frequent observed degeneration in rotator cuff and BT in DM group (*p* < 0.002). Increased rate of SST tears in DM group (*p* < 0.03). More effusions in SAD (*p* < 0.03) and tenosynovitis in BT (*p* < 0.001) in the DM group. Pathological findings prevalent in both groups, but not related with duration of DM
Balci et al. (1999) [28]	Cross sectional	297 subjects with DM type Il Group 1: 86 subjects DM type Il and adhesive capsulitis (age 59.23 ± 24 Group 2: 211 subjects DM type Il without adhesive capsulitis (age 53.6 ± 10.2)	/p/ROM (goniometer): ABD, IR, Blood samples	FS associated with reduced/p/ROM (*p =* 0.006), the age (*p =* 0.000), and duration of DM (*p* = 0.03).
Cole A et al. (2009) [8]	Cross sectional	3206 subjects (of which 682 with shoulder pain and/or stiffness; 221 with DM (age 20–95; median 45)	ROM (inclinometer, visual): SPADI-questionnaire Blood samples	DM patients (or elevated HbA1c levels) had higher prevalence of shoulder pain and/or stiffness (*p* = 0.02).
Czelusniak et al. (2012)) [29]	Cross sectional	150 subjects with DM type Il (age 60.5 ± 12)	UCLA-m rating scale Blood samples	Pain present in 63,4% and dysfunction in 53.4%. No association between HbA1c and joint function, except for/a/FLROM and fasting blood glucose (*p* = 0.026)
Handa et al. (2003) [30]	Case control	Group 1: 14 subjects with rotator cuff disease and DM type Il (age 56.8 ± 7.2 yrs) Group 2: 53 subjects with rotator cuff disease without DM (age 54.9 ± 8.5 yrs)	Synovia specimens from subacromial bursa	Symptom duration not different between groups. Synovial proliferation more frequent in DM vs. non DM (*p* =0.0329) Shoulder joint contracture more frequent in DM vs. non DM (*p* = 0.0045)
Kang et al. (2010) [31]	case control	Group 1: 80 subjects with DM type Il and chronicshoulder pain (age 62.6) Group 2: 339 controls without DM type Il and chronic shoulder pain (age 56.9)	US evaluation rotator cuff	No difference in RC tearsor calcifying tendinopathy between DM vs non DM (*p* =ns)
Lee et al. (2015) [32]	Cross sectional	107 subjects with FS (age 46–68)	Diabetes status, Kcap /p/ROM (goniometer): FL, ABD, ER	Kcap: DM = nDM (*p* = ns) Kcap was negatively correlated with/p/ROM (*p* < 0.005)
Mavrikakis et al. (1989) [33]	Case control	Group 1: 824 subjects with DM type Il (age 66.1 yrs) Group 2: 320 non DM controls matched for age and sex (age 65.7 yrs)	X rays of the shouldersblood sample	Calcific shoulder periarthritis in DM> non DM (*p* < 0.001) Serum mean values: DM = non DM (*p* = ns)
Mavrikakis et al. (1991) [34]	Case control	Group 1: 900 subjects with DM type Il (age 36–93 yrs) Group 2: 350 non DM controls matched for age and sex (age 34–87 yrs)	X rays of the shoulders blood sample	3× more frequent calcific shoulder periarthritis in DM vs. non DM, associated with longstanding/poorly controlled DM, hypercholesterolemia, and hypertriglyceridemia.
Ramchurn et al. (2009) [35]	Cross sectional	Group 1: 96 subjects with DM (46 type I & 50 with type Il) Group 2: 100 controls	HAQ health assessment questionnaire) Blood sample	Shoulder capsulitis (25%), carpal tunnel syndrome (20%), tenosynovitis (29%), limited joint mobility (28%) and Dupuytrens contracture (13%) more prevalent in DM vs. non DM (*p* = 0.02); Mean HbA1c was higher in patients with combined shoulder and hand problems (9.1%) than in those with no upper limb problems (8.0%) (*p* = 0.018). No differences between type 1 and 2.
Salek et al. (2010) [36]	Case control	Group 1: 30 subjects with DM type Il with FS Group 2: 30 matched type Il DM without frozen shoulder	Blood sample	Fasting blood sugar (*p* = 0.012) and blood sugar 2 h after breakfast (*p* < 0.01), HbA1C (*p* < 0.05) and serum triglyceride levels (*p* < 0.001) were elevated in group 1 vs. group.
Schulte et al. (1993) [37]	Cross sectional	Group 1: 70 IDDM (age 38.4 yrs +/− 12.8) Group 2: 70 non DM matched controls (age 40.1 yrs +/− 13.3)	/p/ROM (goniometry): FL, EXT, ADD, ER, IR	In general, 6.1% lesser shoulder mobility in DM vs. non DM (*p* < 0.01)
Shah et al. (2015) [38]	case control	Group 1: 26 subjects with DM type Il (age 64.5) Group 2: 26 matched non DM (age 64.2)	SIF, ultrasound evaluation, /a/ROM (Flock of Birds), Shoulder FL strength (dynamometer), DASH.	The mean SIF measure was higher in DM vs. non DM controls (*p* = 0.047). The BT and SST were 47% and 31% thicker (*p* < 0.001), respectively, in DM vs. non DM. Reduced shoulder elevation and ER in DM vs. non DM (*p* < 0.01). Shoulder FL strength was reduced by 27% (*p* = 0.004) in DM vs non DM. DM showed higher disabilities (DASH) than non DM (*p* < 0.01).
Shah et al. (2015) [39]	Case control	Group 1: 26 subjects with DM type Il (age 64.5) Group 2: 26 matched non DM (age 64.2)	/a/ROM (Flock of Birds), SPADI, DASH.	DM showed higher pain and disabilities (SPADI & DASH) vs. non DM (*p* < 0.01). Decreased shoulder EL and ER in DM vs. non DM (*p* < 0.05). No between groups difference in scapular upward rotation, or shoulder IR (*p* > 0.05)
Siu et al. (2013) [40]	Case control	Group 1: 23 with DM; Group 2: 45 non DM. All subjects had with rotator cuff tearing based on MRI or sonographic findings.	Sum of ROM deficit score, Constant score, VAS score, subacromial synovial fluid collection	DM had increased subacromial IL-1β levels (*p* = 0.048), increased Sum of ROM deficit (*p* < 0.001) and increased VAS scores (*p* = 0.022) and lower Constant scores (*p* < 0.001) than non DM.

Abbreviations: yrs = years; DM = diabetes mellitus; ID = insuline dependent; NID = non-insuline-dependent; ROM = range of motion; BT = biceps tendon; SST = supraspinatus tendon; SScT = Subscapularis tendon; IST = infraspinatus tendon; SAD = subacromial-subdeltoid bursa; SPADI = shoulder pain and disability index; DASH = Disabilities of the hand, arm and shoulder; VAS = Visual Analogue Scale; FL = flexion; EL = elevation; EXT = extension; ER = external rotation; IR =internal rotation; ABD = abduction; US = ultrasound; Kcap = capsular stiffness; ns = non-significant; SIF = skin intrinsic fluorescence; IL = interleukin.

**Table 2:** Methodological assessment using the Newcastle Ottawa quality assessment Scale (NOS).

Studies	ltems										Quality
	1	2	3	4	5	6	7	8	9	Score	
Abate et al. (2011) [26]	+	+	+	+	+	+	+	+	+	9/9	B
Abate et al. (2010) [27]	+	+	+	+	+	+	+	+	+	9/9	B
Handa et al. (2003) [30]	+	+	−	+	+	−	+	+	+	7/9	B
Kang et al. (2010) [31]	+	+	−	−	+	+	−	+	+	6/9	B
Kim et al. (2013) [41]	−	+	−	+	−	−	+	+	−	5/9	B
Mavrikakis et al. (1989) [33]	+	+	+	+	+	+	+	+	+	9/9	B
Mavrikakis et al. (1991) [34]	+	+	+	+	+	+	+	+	+	9/9	B
Salek et al. (2010) [36]	+	+	+	+	−	−	+	+	+	7/9	B
Sattar et al. (1985) [42]	+	+	−	−	−	−	+	+	+	5/9	B
Shahet al. (2015) [38]	−	+	+	+	+	+	+	+	+	8/9	B
Shah et al. (2015) [39]	−	+	+	+	+	+	+	+	+	8/9	B
Siu et al. (2013) [40]	−	+	−	+	−	−	+	+	+	6/9	B
Selection 1: Is the case definition adequate? 2: Representativeness of the cases 3: Selection of controls. 4: Definition of controls											
Comparability 5+6: Comparability of cases and controls on the basis of the the design or analysis											
Exposure 7: Ascertainment of exposure 8: Same method of ascertainment for cases and controls 9: Non-Responserate											

**Table 3:** Methodological assessment using specific criteria described in the AHRQ to assess the risk of bias for benefits for cross-sectional studies.

Studies	ltems										Quality
	1	2	3	4	5	6	7	8	9	Score	
Balci et al. (1999) [28]	+	+	+	−	−	+	+	−	+	6/9	C
Bridgman et al. (1972) [43]	+	−	+	−	−	+	+	−	+	5/9	C
Cole et al. (2009) [8]	+	+	+	+	−	+	+	+	+	8/9	C
Czelusniak et al. (2012) [29]	+	−	+	+	−	+	+	−	+	6/9	C
Escalente et al. (1999) [44]	−	+	+	−	−	−	+	+	+	5/9	C
Lee et al. (2015) [32]	+	+	+	+	−	+	+	+	+	8/9	C
Ramchurn et al. (2009) [35]	−	+	−	+	+	+	+	−	+	6/9	C
Schulte et al. (1993) [37]	+	+	+	+	−	+	+	+	+	8/9	C
Withrington et al. (1985) [45]	−	−	−	−	−	+	−	−	+	2/9	C
Yian et al. (2012) [46]	+	+	+	+	−	+	+	+	+	8/9	C
Selection bias 1: Did the study apply inclusion/exclusion criteria uniformly to all comparison groups? 2: Does the design or analysis control account for important confounding and modifying variables through matching, stratification, multivariable analysis, or other approaches?											
Performance bias 3: Did researchers rule out any impact from a concurrent intervention or an unintended exposure that might bias results?											
Attrition bias 4: If attrition (overall or differential non response, dropout, loss to follow-up, or exclusion of participants) was a concern, were missing data handled appropriately (e.g., intention-to-treat analysis and imputation)?											
Detection of bias 5: Were the outcome assessors blinded to the intervention or exposure status of participants? 6: Were interventions/exposures assessed/defined using valid and reliable measures, implemented consistently across all study participants? 7: Were outcomes assessed/defined using valid and reliable measures, implemented consistently across all study participants? 8: Were confounding variables assessed using valid and reliable measures, implemented consistently across all study participants?											
Reporting bias 9: Were the potential outcomes prespecified by the researchers? Are all prespecified outcomes reported?											

## Data Availability

Not applicable.
